# The Spirit of Competition: To Win or Not To Win

**DOI:** 10.1371/journal.pcbi.1003413

**Published:** 2013-12-26

**Authors:** Teresa Szczepinska, Wataru Iwasaki, Thomas Abeel

**Affiliations:** 1 Institute of Genetics and Biotechnology, Faculty of Biology, University of Warsaw, Warsaw, Poland; 2 Institute of Biochemistry and Biophysics, Polish Academy of Sciences, Warsaw, Poland; 3 Genetic Research Section, Center for Earth Surface System Dynamics, Atmosphere and Ocean Research Institute, the University of Tokyo, Kashiwa, Chiba, Japan; 4 Broad Institute of MIT and Harvard, Cambridge, Massachusetts, United States of America; 5 VIB Department of Plant Systems Biology, Ghent University, Belgium

## Abstract

A competition is a contest between individuals or groups. The gain is often an award or recognition, which serves as a catalyst to motivate individuals to put forth their very best. Such events for recognition and success are part of many International Society for Computational Biology (ISCB) Student Council Regional Student Groups (RSGs) activities. These include a popular science article contest, a Wikipedia article competition, travel grants, poster and oral presentation awards during conferences, and quizzes at social events. Organizing competitions is no different than any other event; they require a lot of hard work to be successful. Each event gives remarkable organizational and social experience for students running it, while at the same time the participants of the competitions are rewarded by prizes and recognition. It gives everybody involved an opportunity to demonstrate their extraordinary talents and skills. Competitions are unique because they bring out both the best and worst in people.

## Competitions in the Context of Science

Science is all about competition. Getting a grant, getting your results published first, getting selected for that top tier journal: it is all extremely competitive. However, competition means different things to different people—for some it is fun, for others it's a way to prove their superiority. Some might find competition silly, but the fact is that we are a competitive species, in virtually everything we do. Competition in a scientific context provides unique opportunities and benefits to the participants.

First of all, competition is an enjoyable way to test your mettle against your colleagues. These competitions give you a chance to brush up on useful skills that are not connected to your field of research. For example, participating in a composition contest will likely improve your writing, either by spending significant effort to make the best entry possible, or by closely observing what the other contestants are doing. Secondly, while participating is more important than winning, you get a shot to win fame and gold. Although gold is out of fashion lately, monetary or other material prizes are still commonplace. The recognition received from winning may be useful to boost your CV just that little bit extra and set you apart from other candidates in your next job search. Winning a competition is definitely a major confidence builder. Being confident in your own abilities is a crucial asset for aspiring students. For those not winning, well, they get an even more important lesson as a budding scientist: how to deal with adversity. Science is 99% failure (or hard work) and 1% luck, so you could say that the losers get the most valuable lesson. Either way, you always gain by participating in a competition. Last, but not least, there are the social interactions. Competitions in the scientific realm tend to be social activities. In many instances you have to work together as a team. Learning to collaborate under the stress of a ticking clock is of critical importance to any scientist. However, even if it is an individual competition, you will probably have to interact socially with your opponents, preferably in a civilized way. After all, the world of computational biology is too small to keep vendettas going. In a way, all these rather friendly forms of competition are a good introduction to the cut-throat competition for grant money later in your career. You and your colleagues are competing over the same pot of grant money, but you still need to be friendly enough to collaborate on other projects.

RSGs have organized many different types of competitions including popular science writing contests, travel fellowships, poster and presentation awards, and quizzes. In this article, we describe some of the experiences we had when organizing these events.

## Popular Science Writing Contests: Competitions Are Valuable, No Matter Who Wins

In 2011, RSG Poland organized a contest in collaboration with a local bioinformatics magazine (www.bioinformatyk.eu), based on making an English version of the magazine's Polish web portal. This magazine focuses on articles in bioinformatics that are easy for students to understand. We invited contributions from all over the world and had 15 submissions, from France, India, Poland, the United Kingdom, and the USA. Two prizes were sponsored by the ISCB Student Council and the Dean of the Biological Faculty at the Adam Mickiewicz University in Poznan, Poland. The entries were judged by an international jury of experts and readers of the magazine. In our opinion, one of the major successes of this contest was the international outreach, which brought together people from three different continents. Participants got a chance to compare their writing abilities with people working in the same field. Moreover, the contest was a lesson on how to make bioinformatics understandable and accessible to a wider audience.

During the summer of 2012, ISCB together with WikiProject Computational Biology announced their Wikipedia Competition, with the goal to improve the coverage on Wikipedia of computational biology articles. The competition was open to students and trainees at any level, and gave them four months to improve a Wikipedia article of their choice in the field of computational biology. The ISCB Student Council took responsibility for reviewing all submitted entries. Using a public and open platform for the competition seemed like a good idea, in particular because any additions would immediately benefit all Wikipedia users. However, there are several challenges that come with having non-contestants running around in the same playground as contestants. Teasing out the contributions made by each contestant during those four months, and identifying edits made by other Wikipedia users, was challenging to say the least. In the end, two dozen contestants competed, and as a result, several new Wikipedia articles were made, and some others were dramatically improved.

## Poster and Presentation Awards: Promoting Excellence

While two examples from the previous section were clearly identified as public competitions, there are many other aspects of science that focus more on the award and less on the competitive process to get there. Among these are the best-poster and best-presentation awards that are included at a variety of meetings. The goal of these awards is to motivate people to work harder on the presentations they are to give, or at least to reward the people who went the extra mile in preparing their presentations. Announcing poster and presentation awards improves the overall quality of the presentations and posters at a meeting, as experienced by some Asian RSGs.

Colleagues from four RSG Asia countries, Japan, Korea, Singapore, and Taiwan, organized the Asian Young Research Conference on Computational and Omic Biology (AYRCOB) in 2008. In AYRCOB, students and young researchers are given the opportunity to present their research in the form of oral and/or poster presentations. The low acceptance rate (less than 20%) makes this extremely competitive; this is well below the acceptance rate of many well-established journals. Furthermore, the committee introduced awards for the best oral and poster presentation, in order to motivate the young participants. Presentations, which came from about ten Asian countries, were judged by distinguished senior researchers who gave invited lectures at the conferences. The competition with colleagues from different countries provided participants with excellent experiences and enduring friendships across the borders.

## Quizzes: Test the Knowledge

In commemoration of the Malaria World Day Symposium, held in Covenant University, Ogun State, Nigeria in 2012, RSG Western Africa conducted a Computational Biology/Bioinformatics scientific quiz for final year students. Participants were tested on their knowledge of Computational Biology and Bioinformatics concepts. The program started with about 46 biology, microbiology, and biochemistry students, from which the best six were picked for the final, public competition, which featured 50 multiple choice questions. Students had the opportunity to choose any of the questions until they answered six questions each. The four top students were given cash awards, with prizes funded by the ISCB Student Council grant.

## Organizing Competitions

How do you organize a competition, and what are the challenges you should expect? Whatever type of competition you wish to organize, there are a few ground rules that will help you run a successful event.

The first step is to announce your competition on time. Timeliness means announcing months in advance, not 48 hours before the closing deadline. You should also regularly remind people of the timeline, including emphasising when submissions are due, and explaining where they should go and when results will be available. Publicize your competition through channels that reach prospective participants, either the society newsletter, notice boards in your local universities, local mailing lists, etc. The opportunities are many, but you have to push the information to potential participants.

Submission-based competitions need authoritative reviewers, who must be lined up in advance. This will make your award or competition more prestigious and will help attract participants. You must ensure sufficient reviewers per submission, without giving reviewers too many submissions to go through. These challenges can be partially addressed by having students or other more junior scientists pre-screen submissions and create a short-list for the final judging panel. Reviewers need to be kept aware of the timeline so that they can schedule the time to complete their reviewing to meet your deadline. Whichever type of competition you're running, the rules have to be crystal clear and the reviewing process should be equally transparent and understandable for everyone involved.

Take into account specific local challenges and opportunities. If your competition involves international participants, you should figure out the logistics of sending prizes abroad or make alternative arrangements. Be sensitive to cultural differences. You might also discover region-specific challenges in reviewing submissions: for example, RSG Asia encountered difficulties in evaluating preliminary work because it was hard to distinguish the contribution of the contestant from the help given by the supervisor. Cultural differences enable you to be creative with which prizes to offer: RSG Western Africa found that a picture of the winners with ISCB directors and invited scientists was an inexpensive, but valued additional prize.

It is important that students take responsibility for the brunt of the organizational work; they will gain from that the most. However, third parties—such as professors, university administrators, private/public companies, and non-profit organizations—can also play an important role. In particular, they can help in gaining recognition and publicity, and in offering prizes and venues. The time professors can offer for reviewing submissions is priceless. A competition can be a valuable lesson for both organizers and participants.

## Conclusion

Due to the competitive nature of science as a whole, competitions provide valuable experience to young, aspiring scientists. Students can learn how to work as a team under stress, how to organize and interact with competitors, and how to deal with success or disappointment. And all this can be achieved in a fun atmosphere.

**About the Authors**

The authors have worked on many aspects of the ISCB Student Council and the Regional Student Group program.

**Teresa Szczepinska** was co-founder and president of RSG-Poland (2009–2012) and served as vice-chair for the ISCB Student Council from January 2011 to January 2013.

**Wataru Iwasaki** was co-founder and secretary of RSG-Japan (2009–2011).

**Thomas Abeel** was co-founder, president (2009), and secretary (2010–2011) of RSG-Europe and served as Student Representative to the ISCB Board of Directors from January 2011 to January 2013.

